# Curcumin reduces malondialdehyde and improves antioxidants in humans with diseased conditions: a comprehensive meta-analysis of randomized controlled trials

**DOI:** 10.1051/bmdcn/2019090423

**Published:** 2019-11-14

**Authors:** Mohammad Alizadeh, Sorayya Kheirouri

**Affiliations:** 1 Nutrition Research Center, Tabriz University of Medical Sciences Tabriz Iran; 2 Department of Nutrition, Tabriz University of Medical Sciences Tabriz Iran

**Keywords:** Curcumin, Curcuma longa, Curcuminoid, Malondialdehyde, Antioxidant, Oxidative stress, Randomized controlled trials, Turmeric

## Abstract

Objective: This systematic review and meta-analysis was conducted to collate the effects of curcumin on MDA and antioxidant markers in individuals with diseased conditions. In this study the research question was “does curcumin supplementation improves oxidative stress and antioxidant defense enzymes in human subjects compared to a group without curcumin supplementation?

Methods: This research included randomized controlled trials published in English in any year, in which intervention with curcumin was compared to either placebo, or standard of care or no intervention. Pubmed, Embase, Cochrane Central, Scopus and Google Scholar were searched. Meta-analysis was performed using RevMan (version 5.3), with standardized mean differences (SMD) and random-effects models.

Results: One hundred twenty-seven titles and abstracts were identified which 17 articles were included for final analysis. The number of participants ranged from 22 to 160 across the included studies. The duration of intervention, dose of curcumin and location of outcomes measurements varied across the studies. Curcumin significantly reduced MDA [SMD −0.46 (95% CI: −0.68 to −0.25)] and increased superoxide dismutase (SOD) [0.82 (0.27 to 1.38)], catalase [10.26 (0.92 to 19.61)], and glutathione peroxidase [8.90 (6.62 to 11.19)] when compared with control group. Subgroup analyses displayed that curcumin could significantly reduce MDA levels with or without use of piperine, however it could increase SOD level in presence of piperine.

Conclusions: These findings suggest that curcumin may be used as an adjunct therapy in individuals with oxidative stress. The administration of piperine with curcumin may enhance the efficacy of curcumin on antioxidant defense system.

**Abbreviations**
ARE: Antioxidant response element
CAT: Catalase
CMA: Comprehensive Meta-Analysis
FOXO: Forkhead box O
GC-MS: Gas chromatography mass spectrometry
GSH: Glutathione
GPX: Glutathione peroxidase
GR: Glutathione reductase
HO-1: Hemeoxygenase-1
Keap1: Kelch ECH associating protein 1
MDA: Malondialdehyde
iNOS: Nitric oxide synthase
NO: Nitrous oxide
Nrf2: Nuclear factor erythroid 2-related factor 2
NF-kB: Nuclear factor-kappaB
PGC-1a: Peroxisome proliferator-activated receptor gamma coactivator 1-a
RCT: Randomized controlled trial
ROS: Reactive oxygen species
GSH: Reduced glutathione
SIRT: Sirtuin
SOD: Superoxide dismutase
TCF/LEF: T cell factor/lymphoid enhancer factor
TBARS: Thiobarbituric acid-reactive substances
TAC: Total antioxidant capacity.

## 1. Introduction

Oxidative stress is a condition in which reactive oxygen species (ROS) are produced in the cells of living beings. Under normal conditions, the concentration of ROS is regulated by internal defense mechanisms including enzymatic and non-enzymatic antioxidants. In living organisms, an increase in the concentration of oxidants compared to antioxidants is termed as oxidative stress, which can impair proteins, lipids, DNA and other components of the cells. Oxidative stress is involved in the initiation and progression of various pathological conditions including diabetes, cancer and neurological disorders [1, 2]. Production of malondialdehyde (MDA), a well-known end product of lipid peroxidation, is up-regulated in response to increased amount of free radicals. Therefore, MDA concentration is a marker of oxidative stress [3]. Increased levels of MDA contribute to the pathogenesis of several metabolic diseases including diabetes [4], cancer [5], cardiovascular events [6], obesity [7] and neurological diseases such as Alzheimer [8] and depression [9].

Antioxidants are molecules that inhibit the production of free radicals and are classified into two types; enzymatic [superoxide dismutase (SOD), glutathione peroxidase (GPx), catalase (CAT), etc.] and non-enzymatic [vitamin C, vitamin E, carotenoids, glutathione (GSH), etc.] [10]. In healthy conditions, the integrated antioxidant systems of organisms are able to scavenge ROS or suppress their detrimental effects. However, during illness, the antioxidant systems are unable to manage the increased amount of ROS. According to a large body of evidence, the concentration of endogenous antioxidants is decreased in various metabolic and neurological diseases [1, 11].

Curcumin is a potent exogenous non-enzymatic antioxidant with diverse pharmacological properties. It is a natural yellow polyphenol compound, derived from Curcuma Longa Rhizome, which possesses the capacity to scavenge free radicals, reduce the generation of ROS and act as strong inhibitor of lipid peroxidation and advanced glycation end products [12–15]. Despite various *in vitro,* animal models and human studies no significant toxicity of curcumin is reported and it is considered generally recognized as safe (GRAS) by United States Food and Drug Administration. Numerous clinical trials have indicated that curcumin inhibits oxidative stress in several chronic diseases [16]. However, it is not frequently used as a therapeutic approach in part due to the fact that information about its advantages have not been generally synthesized and disseminated. Recently, a meta-analysis study addressed curcumin and oxidative stress [17]. However, the antioxidant defense system has not been measured comprehensively and the effects of curcumin on several antioxidant variables such as GSH, CAT, TAC and GR have not been intensively reviewed. This study was therefore conducted to collate the effects of curcumin on oxidative and antioxidant markers, with particular emphasis on MDA, SOD, CAT, total antioxidant capacity (TAC), GSH, GPx and glutathione reductase (GR) in diseased individuals under conditions of oxidative stress.

## 2 Methods

### 2.1. Search strategy and selection criteria

This meta-analysis was based on the preferred reporting items for systematic review and meta-analysis protocols (PRISMA-P) 2015 statement. The pre-defined inclusion criteria were as follows: published full text randomized controlled trials (RCTs) in English, published at any point until January 2019, and all of the studies comparing curcumin treatment (in any form) with a placebo intervention or with standard therapy. Studies assessing the effect of curcumin in combination with other plants were excluded. Markers of oxidative stress (MDA) and of the antioxidant defense system (SOD, CAT, GPx, GSH, TAC, GR) were the outcomes of interest. The databases searched were Pubmed, Embase, Cochrane Central, Scopus and Google Scholar. Keyword and MeSH searches for [curcumin or curcuminoid or curcuma longa or turmeric] [in title] and [oxidative stress or reactive oxygen species or free radicals or antioxidant defense or malondialdehyde or superoxide dismutase or oxidative damage or pro-oxidant] [in title / abstract] were carried out in each database. Google and Google Scholar cites were hand searched for additional studies and the reference lists of the relevant reviews were checked. The PICO (Patient/Population; Intervention; Comparator; Outcome) question was as follows: in humans with diseased conditions (P), does curcumin supplementation (I) compared to placebo or standard care (C), improves oxidative stress and antioxidant defense enzymes (O)? Description of the PICOS strategy is shown in Table 1.

**Table 1:** Description of the PICOS strategy.

Population	Patients under oxidative stress conditions
Intervention	Any form of curcumin supplementation
Comparator	Defined as placebo intervention or standard therapy
Outcome	MDA and antioxidant defense enzymes (SOD, CAT, TAC, GSH, GPx, and GR)
Setting	Serum, plasma and mucosa

Superoxide dismutase (SOD); Total antioxidant capacity (TAC); Malondialdehyde (MDA); Reduced glutathione (GSH); Catalase (CAT); Glutathione reductase (GR); Glutathione peroxidase (GPX); Glutathione (GSH).

### 2.2. Data extraction and quality assessment

All relevant titles and abstracts were transferred to Endnote Web, sorted into separate duplicated references and then screened based on a critical analysis of title and article summary. Each article text was then individually reviewed for evaluation of the eligibility of the article and for data extraction. A third investigator was discussed when there was disagreement on an article, and the authors were emailed when insufficient information about the data was determined. Corel software [CorelDRAW Graphics Suite 2017 (64-Bit)] was used to extract the numeric values of data which had been presented as a figure. The quality of the studies included were evaluated using Review Manager, version 5.3 (Cochrane Collaboration), focusing on bias in selection, performance, detection, attrition, and reporting. In addition, bias in the matching of control and treatment groups at entry regarding of participants number; type and dose of medications, type of therapy, health status, age and sex was examined.

### 2.3. Meta-analysis

Of the studies included, data from after treatment (not baseline) from the curcumin and control groups were compared as mean ± standard deviation (SD) in the meta-analysis. SD = standard error of the mean × n (number of participants used for analyses) was used as the formula to estimate SD. The SI Conversion Calculator was used to obtain the desired units. MDA and TAC levels were collated in μmol/l; SOD, CAT, GPx and GR in U/ml; and GSH in μg/*ml*.

If a study had more than one arm of curcumin treatment, each arm was considered as a single study. Review Manager (version Cochrane Collaboration) was used for meta-analysis, and effect size was calculated as the weighed mean difference with a 95% confidence interval. Funnel plots and Egger’s weighted regression test were used to explore potential publication bias using Comprehensive Meta-Analysis (CMA) V3 software (Biostat, NJ). Meta-regression and subgroup analyses were conducted to investigate the role of factors possibly affecting heterogeneity, such as the dosage of curcumin supplements, the duration of treatment, the addition of absorption enhancers and the age of participants. Random-effects models were used due to the heterogeneity of participants. All of the outcomes were continuous measures. Multiple sensitivity analyses were carried out to identify if any of the findings were affected by the studies not included in the metaanalysis.

## 3. Results

**Fig. 1:**
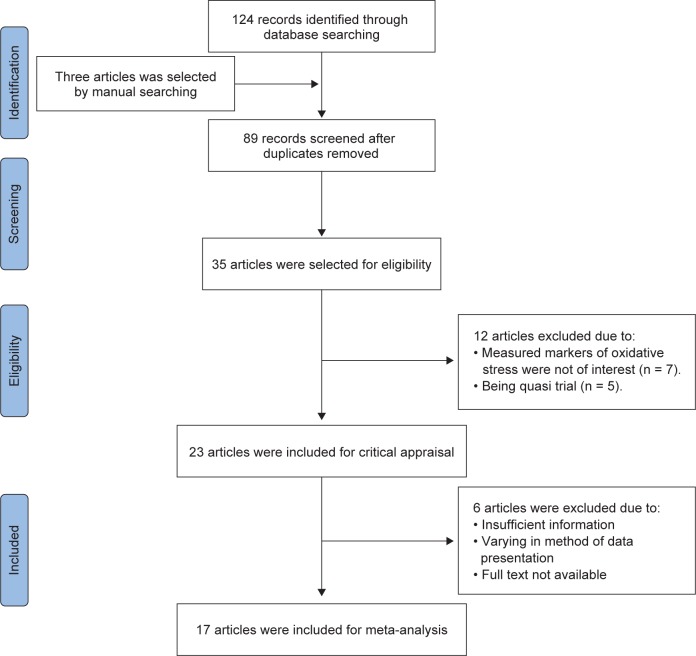
Flow diagram of the study.

As shown in Fig. 1, one hundred and twenty-four records were found through database searches and three articles were identified by manual searching (one from Google, one from Google Scholar and one by reference searching). Eighty nine articles were screened after the duplicates were removed, of which 35 articles were eligible. Twelve articles were then excluded due to undesired markers of oxidative stress (n = 7) and being quasi trial (n = 5). Thus, 23 articles were remained for critical appraisal. At this phase, 6 articles were excluded due to lack of sufficient information, variation in method of data presentation and unavailability of the full text. At the end, 17 articles were included for meta-analysis. Of those, some articles were excluded for certain outcomes as follows: Judaki *et al.* [18] study for GPx; Pakfetrat *et al.* [19] study for GPx, CAT and GR; Panahi *et al.* [20] study for TAC; Sudheeran *et al.* [21] study for SOD, CAT and GSH outcomes since the data had been presented as g or mg protein.

**Fig. 2:**
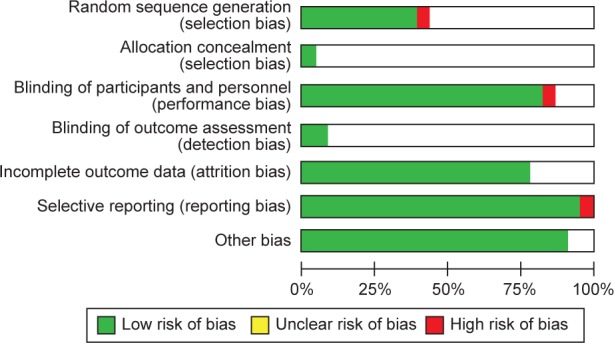
Risk of bias graph: review authors’ judgements about each risk of bias item presented as percentages across all included studies.

**Fig. 3:**
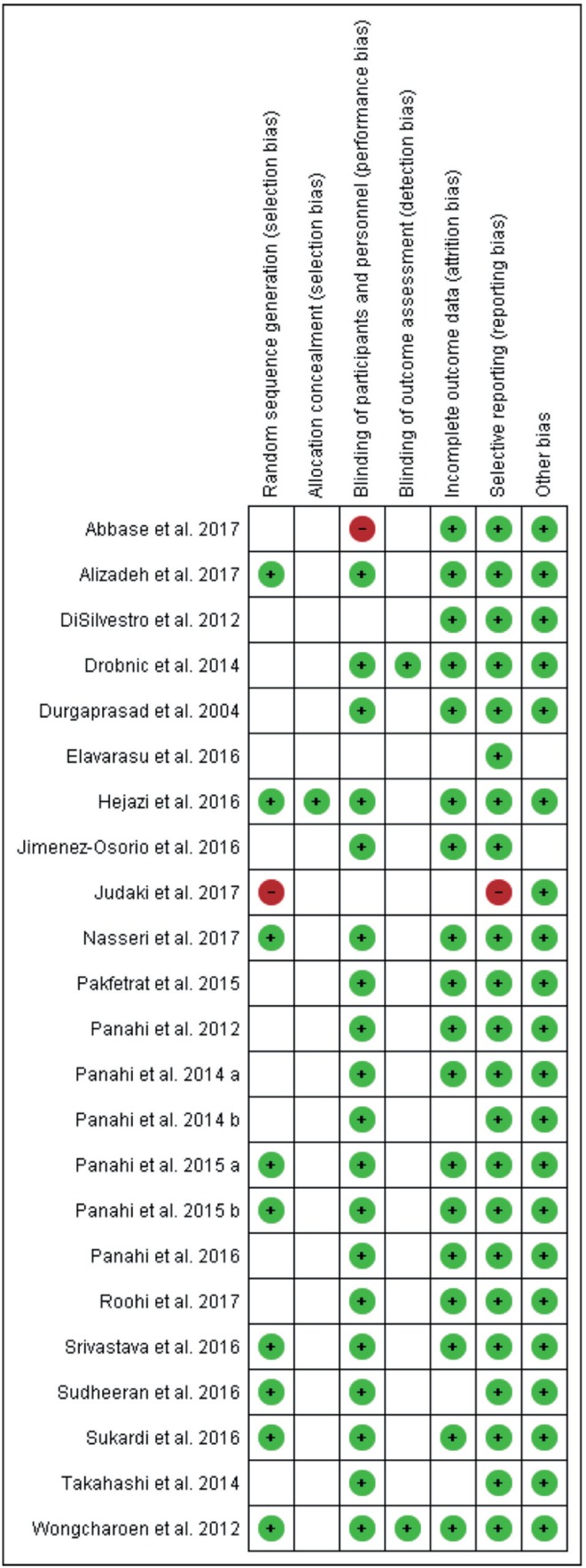
Risk of bias summary: review authors’ judgements about each risk of bias item for each included study.

**Fig. 4:**
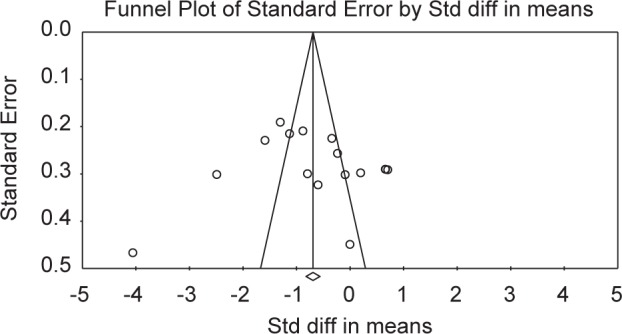
Funnel plot of the studies included.

As shown in Fig. 2, reporting bias was not common among the studies. Allocation concealment and masking of outcome assessment was not stated by most of the studies. As shown in Fig. 3, the quality of the studies included differed and some of the studies did not contain sufficient details to evaluate all aspects of quality. The studies by Elavarasu *et al.* [22] and Takahashi *et al.* [23] did not get eligible criteria for inclusion. Publication bias of the studies included was shown by the Cochrane tool (Figures 2 and 3) and funnel plot (Fig. 4). According to Egger’s test there was no publication bias *(p* = 80) for MDA.

The characteristics of the investigations included are summarized in Table 2. The included studies were published from 2012 to 2017, and the number of participants varied from 22 to 160. Further, the populations studied were with various health conditions *(e.g.* healthy, P-Thalassemia, chronic gastritis, etc.). The duration of treatment varied from 1 week to 4 months across the studies.

**Table 2:** Characteristics of the included studies.

Author year (ref.)	Country	Number (sex)	Age	Type of disease	Duration of treatment	Control (number at entry)	Treatment group (number at entry)	Time of measures	Type of study
Abbase *et al.* 2017^24^	Iraq	40 (M/F)	42.62 ± 13.84	Peptic ulcer disease	14 days	Standard triple therapy (N = 19)	Curcumin (1500 mg/day) + standard triple therapy (N = 21)	Baseline and after 6 weeks	RCT
Alizadeh *et al.* 2017^25^	Iran	60 (M)	30.27 ± 3.99	Infertile oligoasthenospermia	10 weeks	Placebo (n = 30)	Curcumin nanomicelle 80 mg/day (n = 30)	Baseline and end of study	RCT
DiSilvestro *et al.* 2012^26^	USA	38 (M/F)	47.5 ± 10.5	Healthy	4 weeks	Placebo (n = 19)	Curcuma Longa root powder 400 mg/day (n = 19)	Before and after treatment	RCT
Hejazi *et al.* 2016^27^	Iran	40 (M)	70.71 ± 8.20	Prostate cancer treated with radiotherapy	3 months	Placebo (n = 23)	Curcuminoids 3 g/day (n = 22)	Baseline and after 3 months	RCT
Jimenez- Osorio *et al.* 2016^28^	Mexico	101 (M/F)	40.55 ± 3.05	Non-diabetic or diabetic proteinuric chronic kidney disease	8 weeks	Placebo groups:	Turmeric (320 mg curcumin /day:	Before and after treatment	RCT
						Non-diabetic proteinuric CKD (n = 26)	Non-diabetic proteinuric CKD (n = 24)		
			55.6 ± 1.55			Diabetic proteinuric CKD (n = 23)	Diabetic proteinuric CKD (n = 28)		
Judaki *et al.* 2017^18^	Iran	100 (M/F)	54.15 ± 16.09	Chronic gastritis	4 weeks	Standard triple therapy (N = 50)	Standard triple therapy +Turmeric tablet (700 mg three times/day) (N = 50)	Before and after treatment	RCT
Nasseri *et al.* 2017^29^	Iran	68 (M/F)	26.79 ± 6.57	P-Thalassemia	12 weeks	Placebo (n = 34)	Curcumin 1000 mg/day (n = 34)	Baseline and after 12 weeks	RCT
Pakfetrat *et al.* 2015^19^	Iran	50 (M)	53.6 ± 14.7	Hemodialysis	8 weeks	Placebo (n = 25)	Turmeric 1500 mg/day (n = 25)	Before and after treatment	RCT
				patients					
Panahi *et al.* 2012^30^	Iran	96 (M)	47.9 ± 9.6	Veterans of the Iraq-Iran war with chronic pruritus	4 weeks	Placebo (n = 50)	Curcuminoids 1 g/day (n = 46) + 5 mg bioperine	Before and after treatment	RCT
Panahi *et al.* 2014 a^31^	Iran	89 (M)	50.97 ± 7.27	Sulfur mustard Iraq-Iran war chronic pulmonary complications	4 weeks	placebo (n = 44)	Curcuminoids 1500 mg/day + piperine 15 mg/day (n = 45)	Before and after treatment	RCT
Panahi *et al.* 2014 b^32^	Iran	80 (M/F)	58.95 ± 15.31	Solid cancer	8 weeks	Placebo (n = 40)	Curcuminoids 900 mg/day (n = 40)	Before and after treatment	RCT
Panahi *et al.* 2015 a^33^	Iran	117 (M/F)	44.13 ± 9.18	Metabolic syndrome	8 weeks	Placebo (n = 58)	Curcuminoids 1 g/day + 10 mg piperine (n = 59)	Before and after treatment	RCT
Panahi et al. 2015 b^34^	Iran	40 (M/F)	57.44 ± 8.91	Knee Osteoarthritis	6 weeks	Placebo (n= 21)	Curcuminoid 1500 mg/day + 15 mg piperine (n= 19)	Before and after treatment	RCT
Panahi *et al.* 2016^20^	Iran	118 (M/F)	42 ± 7.5	Type 2 diabetes	8 weeks	Placebo	Curcuminoids 1000 mg/day + piperine 10 mg	Before and after treatment	RCT
Roohi *et al.* 2017^35^	Iran	22 (M)	24.85 ± 2.2	Active healthy males	1 week	Placebo (n = 11)	Curcuminoids 90 mg (n = 11)	Before and after treatment	RCT
Srivastava *et al.* 2016^36^	India	160 (M/F)	50.25 ± 8.35	Osteoarthritis of knee	4 months	Placebo (n = 82)	Curcuma longa extract 1000 mg/day (n = 78)	At day 0, 60, and 120	RCT
Sudheeran *et al.* 2016^21^	India	60 (M/F)	33 ± 7	Occupational stress- related anxiety and fatigue	30 days	Placebo (n = 20)	Formulated curcuminoids 1000 mg/day (n = 20)	Before and after treatment	RCT
							Standard curcuminoids (782 mg curcumin) (n = 20)		

Male (M); female (F); randomized controlled trial (RCT); Age was expressed as mean ± SD or range.

Curcumin had been used in various forms and the dose varied from 80 mg to 4 g/day among the studies. The comparators generally had either the same condition as the curcumin group but received a placebo or routine care. The duration and timings were mostly similar between the control and intervention groups. None of the studies reported any adverse effects for curcumin supplementation.

As shown in Table 3, the studies measured various indicators of oxidative stress and antioxidant defense markers; with measurements taken from areas including serum, plasma, gastric mucosa, gingival crevicular fluid to erythrocytes. MDA was measured mainly by using the thiobarbituric acid reactive substances (TBARS) method.

**Table 3:** Extracted data from the included trials.

Author Year (ref.)		Measured outcomes	Placebo		Curcumin		unit	Method of measure	Data presented as:
			before	after	before	after			
Abbase *et al.* 20 1 7^24^	Serum	TAC	50.7 ± 45	47.63 ± 25.27	37.06 ± 12.66	40.31 ± 13.24	ng/ml	Elisa	Mean ± SD
Alizadeh *et al.* 20 1 7^25^	Serum	MDA	1.05 ± 0.1	1.08 ± 0.1	0.96 ± 0.09	0.73 ± 0.07	μmol/l	TBARS method	Mean ± SD
		TAC	1.36 ± 0.08	1.35 ± 0.09	1.24 ± 0.07	1.95 ± 0.05	μmol/l	Colorimetry	Mean ± SD
DiSilvestro		CAT	28 ± 4	27 ± 4	29 ± 2	50 ± 7	nmol/ml	kit	Mean ± SD
*et al.* 2012^26^	Plasma	SOD	4658 ± 405	4748 ± 458	4798±401	4807 ± 445	U/ml	Spectrophotometry	Mean ± SD
		GPx			14.6 ± 6.3	15.4 ± 6.2		ELISA	Mean ± SD
Hejazi *et al.* 20 1 6^27^	Plasma	TAC	9.1 ± 2.3	10.6 ± 1.3	10.7 ± 2.0	12.8 ± 2.1	U/mL	Assay kit	Mean ± SD
		SOD	195.2 ± 43.1	215.2 ± 83.5	226.1 ± 143.1	189.4 ± 115.4	U/L		Mean ± SD
		CAT	134.4 ± 62.7	134.8± 60.2	139.7 ± 110.2	111.2 ± 80.9	U/L		Mean ± SD
		GPx	125.9 ± 17.1	126.8± 12.0	123.4 ± 15.4	125.6 ± 13.4	U/mL		Mean ± SD
Jimenez- Osorio *et al.* 20 1 6^28^	Plasma Diabetic	MDA	3.66 ± 0.42	2.59 ± 0.26	3.44 ± 0.37	2.83 ± 0.24	nM	Spectrophotometry	Mean ± SE
		GSH	3.49 ± 0.37	3.029 ± 0.24	2.70 ± 0.24	3.05 ± 0.24	nM		Mean ± SE
		GR	0.107 ± 0.009	3.127± 0.01	0.116 ± 0.01	0.108 ± 0.008	U/mL		Mean ± SE
		GPX	0.20 ± 0.01	0.20 ± 0.01	0.22 ± 0.01	0.18 ± 0.02	U/mL		Mean ± SE
Jimenez- Osorio *et al.* 20 1 6^28^	Plasma Non-diabetic	MDA	3.07 ± 0.39	3.05 ± 0.31	4.06 ± 0.37	2.94 ± 0.22	nM	Spectrophotometry	Mean ± SE
		GSH	2.56 ± 0.28	3.03 ± 0.15	3.84 ± 0.42	3.71 ± 0.33	nM		Mean ± SE
		GR	0.11 ± 0.008	0.118 ± 0.01	0.118 ± 0.007	0.126 ± 0.01	U/mL		Mean ± SE
		GPX	0.22 ± 0.01	0.23 ± 0.04	0.23 ± 0.01	0.28 ± 0.06	U/mL		Mean ± SE
Judaki *et al.* 2017^18^	Gastric mucosa	MDA	2.63 ± 0.42	2.92 ± 0.24	2.47 ± 0.33	2.33 ± 0.70	μmol/L		Mean ± SD
		TAC	2.52 ± 0.37	2.50 ± 0.60	2.04 ± 0.47	1.94 ± 0.32	μmol/L		Mean ± SD
Nasseri *et al.* 20 1 7^29^	Serum	MDA	38.70 ± 12.24	39.90 ± 12.37	41.03 ± 14.96	37.12 ± 11.70	μmol/L	Colorimetric assay	Mean ± SD
		TAC	191.46 ± 76.16	196.10 ± 72.57	187.09 ± 82.26	207.96 ± 91.72	μmol/L		Mean ± SD
		CAT	6.20 ± 3.73	6.01 ± 3.87	5.68 ± 3.95	5.81 ± 3.76	μ/mL		Mean ± SD
Pakfetrat *et al.* 2015^19^	Plasma	MDA	8.6 ± 1.4	7.5 ± 1.1	7.5 ± 2.5	6.0 ± 2.4	nmol/mL	Colorimetric	Mean ± SD
Panahi *et al.* 2012^30^	Serum	SOD	58.01 ± 12.17	57.01 ± 8.84	57.68 ± 12.67	65.85 ± 13.84	μKat/l	kits	Mean ± SD
		GPX	57.34 ± 9.67	56.34 ± 11.67	63.68 ± 9.34	74.68± 21.34	μKat/l		Mean ± SD
		CAT	663.47 ± 66.35	656.63 ± 54.51	647.80 ± 87.68	832.33 ± 154.53	μKat/l		Mean ± SD
Panahi *et al.* 2014 a^31^	Serum	GSH	8.91 ± 1.86	11.33 ± 4.32	10.90 ± 2.77	21.02 ± 4.24	μg/mL	Spectrophotometry	Mean ± SD
		MDA	24.38 ± 3.21	22.47 ± 4.92	25.38 ± 3.61	11.84 ± 3.51	nmol/mL		Mean ± SD
Panahi *et al.* 2014 b^32^	Serum	SOD	0.70 ± 0.19	0.80 ± 0.24	0.75 ± 0.33	2.43 ± 0.62	U/mL	Spectrophotometry	Mean ± SD
		CAT	10.21 ± 1.76	9.57 ± 1.71	14.08 ± 3.13	27.93 ± 4.49	U/mL		Mean ± SD
		GSH	8.85 ± 1.87	11.74 ± 4.25	10.69 ± 3.08	20.96 ± 3.40	μg/mL		Mean ± SD
		MDA	24.38 ± 3.18	25.44 ± 4.75	25.53 ± 3.75	20.23 ± 21.32	nmole/mL		Mean ± SD
Panahi *et al.* 2015 a^33^	Serum	SOD	1.70 ± 0.41	1.98 ± 0.29	1.47 ± 0.31	2.41 ± 0.37	U/mL	Spectrophotometry	Mean ± SD
		MDA	19.56 ± 2.73	20.16 ± 3.11	19.38 ± 3.08	15.62 ± 2.59	nmole/mL		Mean ± SD
Panahi *et al.* 2015 b^34^	Serum	SOD	4.13 ± 0.36	3.76 ± 0.44	4.02 ± 0.29	6.94 ± 0.91	U/mL	Spectrophotometry	Mean ± SE
		GSH	3.43 ± 0.22	3.41 ± 0.43	3.67 ± 0.19	5.06 ± 0.62	μg/mL		Mean ± SE
		MDA	23.11 ± 0.40	20.62 ± 0.99	23.00 ± 0.55	17.73 ± 1.19	nmol/mL		Mean ± SE
Panahi *et al.* 20 1 6^20^	Serum	TAC	3.17 (2.42-3.80)	2.63 (2.24-3.20)	3.17 (2.65-3.89)	3.84 (2.43-4.30)	nmol/mg	Colorimetry	Median (IQR)
		SOD	3.39 ± 1.01	2.96 ± 0.78	3.46 ± 0.99	3.86 ± 0.76	U/mL	Spectrophotometry	mean ± SD
		MDA	3.74 ± 1.32	3.88 ± 0.98	3.90 ± 1.06	3.05 ± 0.91	nmol/mL		mean ± SD
Roohi *et al.* 2017^35^	Plasma	TAC	207.21 ± 14.41	190.09 ± 13.51	190.99 ± 12.61	322.52 ± 21.62	μmol/L	Spectrophotometer	Mean ± SE
		GSH	1.36 ± 0.05	1.51 ± 0.10	1.26 ± 0.029	1.90 ± 0.17	μmol/L		Mean ± SE
		MDA	0.96 ± 0.04	0.97 ± 0.01	0.99 ± 0.026	0.97 ± 0.005	μmol/L	TBARS method	Mean ± SE
Srivastava *et al.* 2016^36^	Serum	MDA	5.15 ± 0.14	4.91 ± 0.11	5.03 ± 0.16	3.69 ± 0.12	nmol/ml	TBARS method	Mean ± SE
Sudheeran *et al.* 2016^21^ (Standard curcumin)	Plasma	SOD	0	0	0	0	U/mL	–	Mean ± SD
		GPx	25.97 ± 3.29	28.60 ± 2.96	33.04 ± 2.17	39.13 ± 1.30	U/mL		Mean ± SD
Sudheeran *et al.* 20 1 6^21^ (Formulated curcumin)	Plasma	SOD	0	0	0	0	U/mL		Mean ± SD
		GPx	25.97 ± 3.29	28.60 ± 2.96	41.85 ± 4.36	67.57 ± 2.61	U/mL		Mean ± SD

Superoxide dismutase (SOD); Total antioxidant capacity (TAC); Malondialdehyde (MDA); Reduced glutathione (GSH); Catalase (CAT); Glutathione reductase (GR); Glutathione peroxidase (GPX); Glutathione (GSH); Thiobarbituric acid-reactive substances (TBARS); Gas chromatography mass spectrometry (GC-MS)

Note: Data of one article was emailed by author. (DiSilvestro *et al.* 2012)

**Fig. 5:**
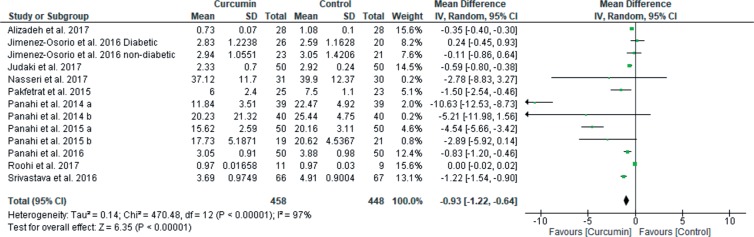
Forest plot of MDA outcome.

**Fig. 6:**
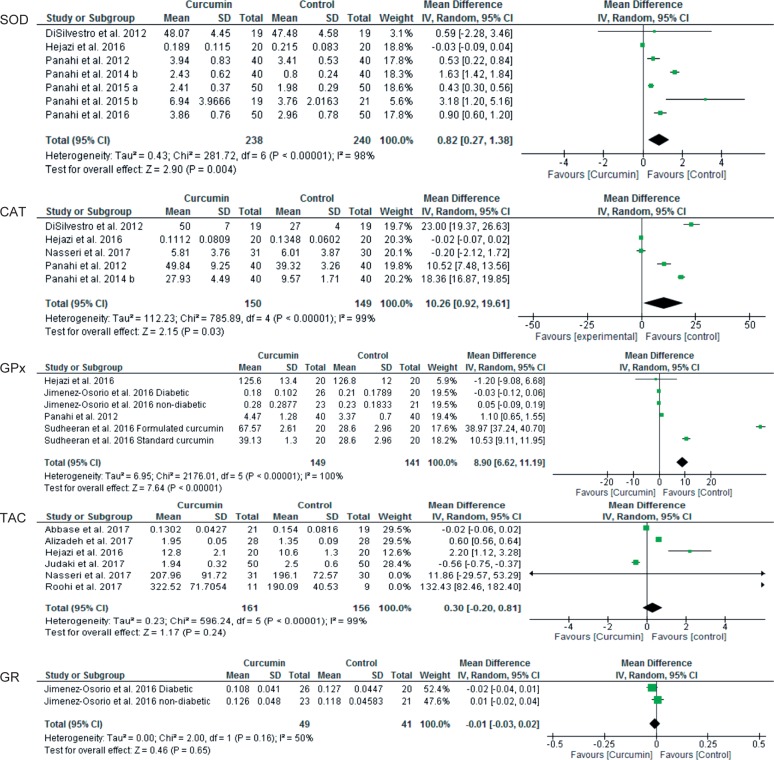
Forest plots of antioxidants markers.

When after intervention levels of MDA and antioxidant enzymes were compared between curcumin and control groups, curcumin treatment significantly reduced MDA levels [13 studies, total 95% CI: −0.93 (−1.22 to −0.64), overall effect: Z = 6.35, *p* < 0.00001, I^2^ = 97%] (Fig. 5); but increased levels of SOD [7 studies, total 95% CI: 0.82 (0.27 to 1.38), overall effect: Z = 2.90, p = 0.004, I^2^ = 98%] (Fig. 6); CAT [5 studies, total 95% CI: 10.26 (0.92 to 19.61), overall effect: Z = 2.15, p = 0.03, I^2^ = 99%] (Fig. 6); GPx [6 studies, total 95% CI: 8.90 (6.62 to 11.19), overall effect: Z = 7.64, *p* < 0.00001, I^2^ = 100%] (Fig. 6). However, curcumin treatment did not significantly impact on levels of TAC [6 studies, total 95% CI: 0.30 (−0.20 to 0.81), overall effect: Z = 1.17, *p* = 0.24, I^2^ = 99%] (Fig. 6) and GR [2 studies, total 95% CI: −0.01 (−0.03 to 0.02), overall effect: Z = 0.46, *p* = 0.65, I^2^ = 50%] (Fig. 6) compared to the control group.

In another analysis, before and after intervention levels of MDA and antioxidant enzymes were compared only in curcumin treated groups. Curcumin treatment significantly reduced MDA levels [13 studies, total 95% CI: −1.41 (−1.76 to −1.06), overall effect: Z = 7.82, p < 0.00001; I^2^ = 97%]; but significantly increased SOD [7 studies, total 95% CI: 0.83 (0.19 to 1.46), overall effect: Z = 2.56, p = 0.01, I^2^ = 98%]; CAT [5 studies, total 95% CI: 9.11 (1.58 to 16.64), overall effect: Z = 2.37, p = 0.02, I^2^ = 99%]; and GPx [7 studies, total 95% CI: 4.04 (3.02 to 5.06), overall effect: Z = 7.78, p < 0.00001, I^2^ = 99%]. However, curcumin treatment did not significantly affect TAC [7 studies, total 95% CI: 0.42 (−0.03 to 0.88), overall effect: Z = 1.81, p = 0.07; I^2^ = 100%]; and GR [2 studies, total 95% CI: 0.00 (−0.02 to 0.02), overall effect: Z = 0.04, p = 0.96, I^2^ = 0%] levels as compared to before intervention values (Data not shown).

**Fig. 7:**
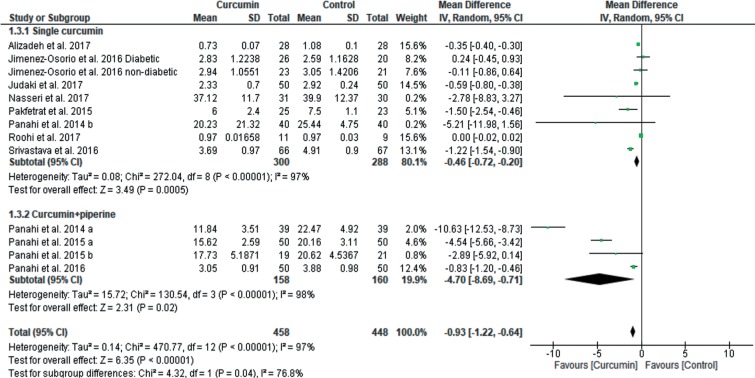
Forest plots of MDA subgroup analysis. (Single curcumin *vs.* Curcumin + piperine)

**Fig. 8:**
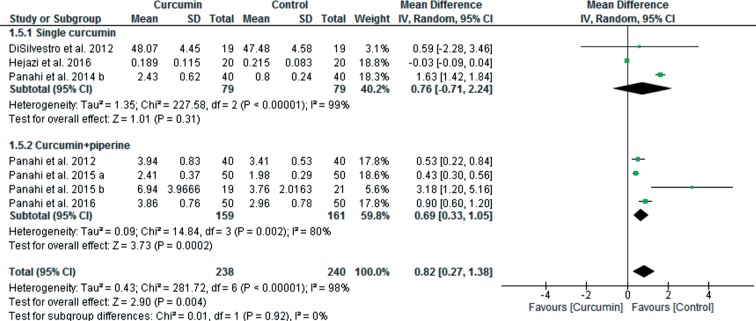
Forest plots of SOD subgroup analysis. (Single curcumin vs. Curcumin + piperine)

Heterogeneity was high for all of the outcomes. The findings of the meta-regression showed that the dose of supplemented curcumin was a factor that significantly influenced heterogeneity (slope: −0.0003; 95% CI: −0.0006 to −0.00007; p = 0.01) for MDA outcome, but age (slope: 0.003; 95% CI: −0.01 to 0.02; p = 0.67) and duration of curcumin supplementation (slope: −0.009; 95% CI: −0.04 to 0.02; p = 0.61) did not impact. Furthermore, according to the poor bioavailability of curcuminoids, some of the studies supplemented curcumin with small doses of pipeline to overcome to this problem. Therefore, subgroup analysis was done to clarify the effect of curcumin supplementation alone compared to curcumin plus piperine supplementation on MDA and SOD levels. As shown in Fig. 7, curcumin significantly reduced MDA levels both without pipeline [9 studies, total 95% CI: −0.46 (−0.72 to −0.20), overall effect: Z = 3.49, *p* = 0.0005, I^2^ = 97%] and with piperine [4 studies, total 95% CI: −4.70 (−8.69 to −0.71), overall effect: Z = 2.31, *p* = 0.02, I^2^ = 98%]. As shown in Fig. 8, supplementation of curcumin along with piperine significantly increased SOD levels [4 studies; total 95% CI: 0.69 (0.33 to 1.05), overall effect: Z = 3.73, *p* = 0.0002; I^2^ = 80%], but without piperine did not significantly increase SOD levels [3 studies, total 95% CI: 0.76 (−0.71 to 2.24); overall effect: Z = 1.01, *p* = 0.31; I^2^ = 99%].

**Fig. 9:**
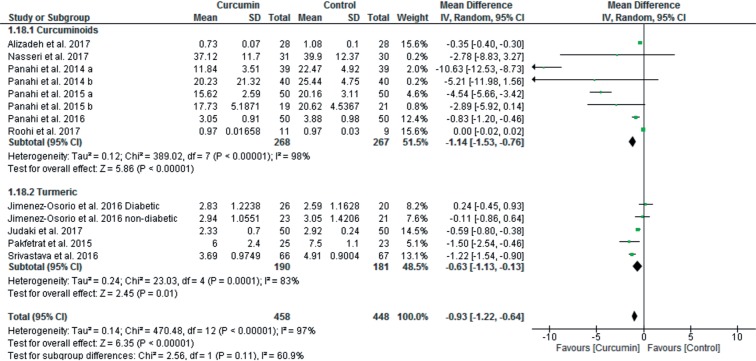
Forest plots of MDA subgroup analysis (Curcuminoids vs. Turmeric powder)

Fourteen studies used curcumin with or without other curcuminoids as supplement in intervention group, which we put all in curcuminoids group, whereas, three studies used turmeric powder. To separate effects of curcuminoids from turmeric powder on variables studied, we did subgroup analysis for MDA. As shown in Fig. 9, either curcuminoids [total 95% CI: −1.19 (−1.53 to −0.76); overall effect: Z = 5.86, *p* < 0.00001; I^2^ = 98%] or turmeric powder [total 95% CI: −0.63 (−1.13 to −0.13); overall effect: Z = 2.45, *p* = 0.01; I^2^ = 83%] significantly reduced MDA level. However, for antioxidant variables, number of studies with turmeric supplementation were too few, for that we did sensitivity test to isolate its effect on the results. Exclusion of studies with turmeric supplementation increased significance of the results for SOD [6 studies, total 95% CI: 0.83 (0.27 to 1.40); overall effect: Z = 2.88, *p* = 0.004; I^2^ = 98%)] and TAC [5 studies, total 95% CI: 0.63 (0.05 to 1.21); overall effect: Z = 2.14, *p* = 0.03; I^2^ = 99%)], but reduced for CAT [4 studies, total 95% CI: 7.15 (−2.62 to 16.92); overall effect: Z = 1.43, *p* = 0.15; I^2^ = 100%)] and GPx [4 studies, total 95% CI: 12.50 (−4.92 to 29.93); overall effect: Z = 1.41, *p* = 0.16; I^2^ = 100%].

## 4. Discussion

**Fig. 10:**
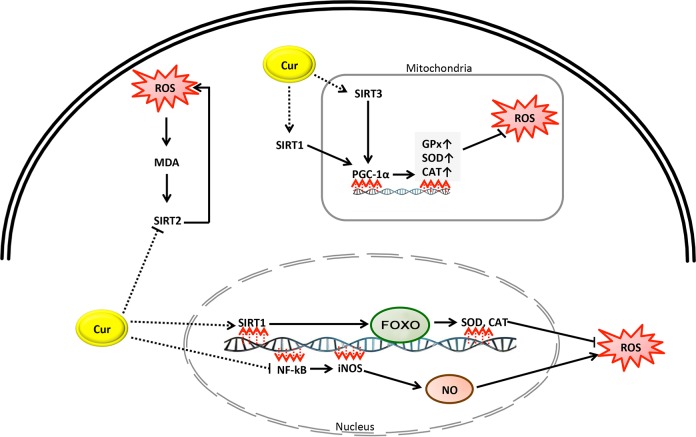
A simplified mechanistic model of curcumin in prevention of oxidative stress through activation or inhibition of sirtuin proteins and NF-KB pathways.

**Fig. 11:**
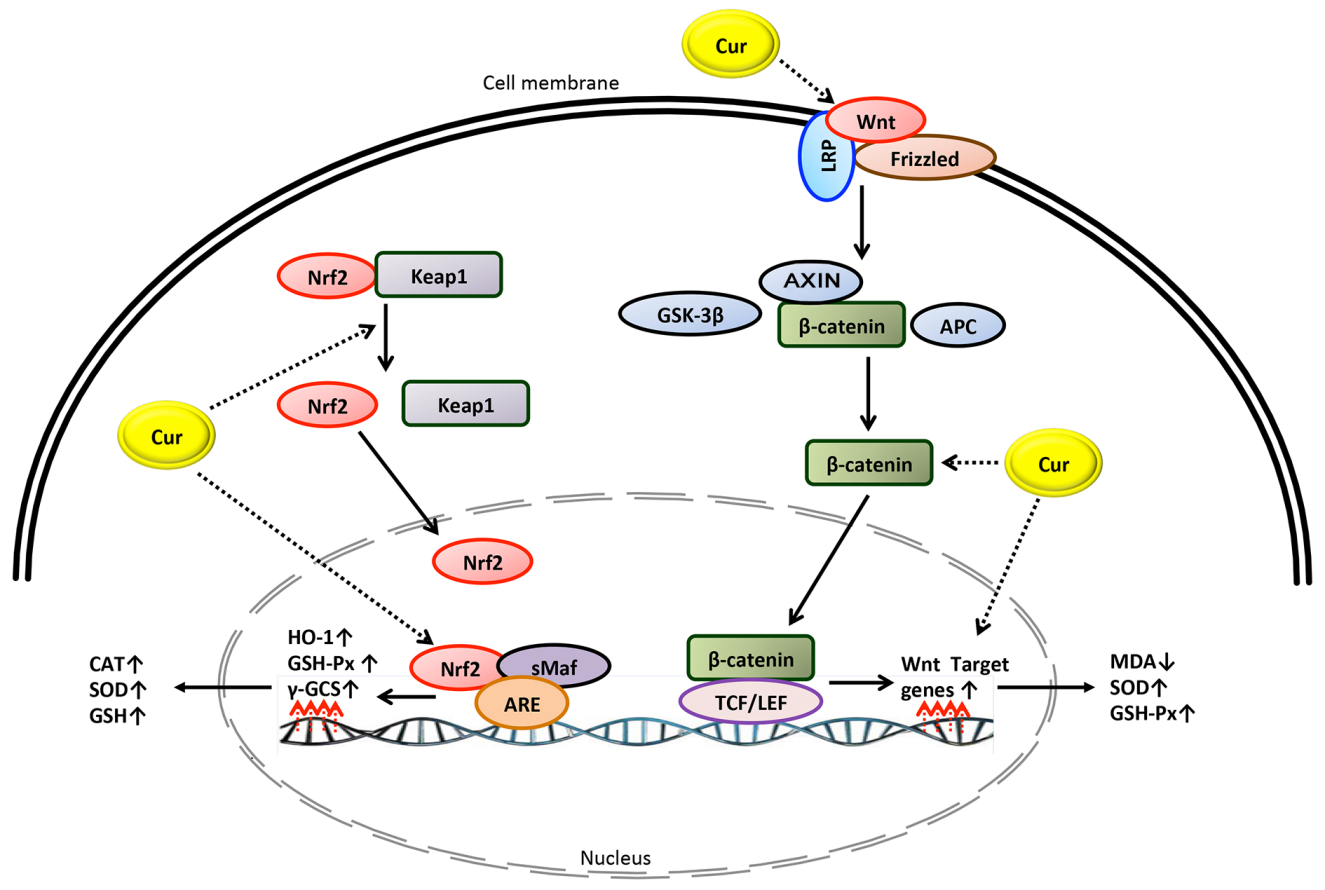
A simplified mechanistic model of curcumin in prevention of oxidative stress through activation of Keap1-Nrf2-ARE and Wnt/β-Catenin signaling pathways.

This study showed that curcumin was effective in reducing MDA and in increasing levels of antioxidants. A large amount of *in vivo,* experimental and human evidence has suggested that curcumin can act as a free radical scavenger and an inhibitor of MDA production. However, the exact mechanism by which curcumin inhibits oxidative stress is unclear. As shown in Fig. 10 and Fig. 11, it is speculated that curcumin prevents oxidative stress through various pathways:

### 4.1. Sirtuins 1, 2 and 3 pathways

The sirtuins (SIRT) are a group of proteins which act as intracellular regulatory proteins, and are involved in multiple cellular processes including aging, resistance to stress, metabolic regulation and transcription. As shown in Fig. 10, the activation or inhibition of sirtuins of 1, 2 and 3 by curcumin may be involved in reducing malondialdehyde and increasing the levels of antioxidants. Various studies suggest that SIRT1 and SIRT3 inhibit oxidative stress in cells [37], whereas SIRT 2 triggers it [38]. Curcumin has been suggested to act as an activator of SIRT1 and SIRT3, but as an inhibitor of SIRT2.

#### 4.1.1. SIRT1

SIRT1 is mostly located in the nucleus and functions by reducing the acetylation of Forkhead box O (FOXO) 3a protein, increasing the binding of FOXO to DNA and activating the FOXO transcription factors that regulate antioxidant genes including SOD and CAT in order to reduce cellular levels of ROS [39–41]. In a review article by Zhang *et al.* [42] it was concluded that SIRT1 inhibits cellular oxidative stress. Further, Miao *et al.* [37] reported that curcumin increased SIRT1 expression. Furthermore, peroxisome proliferator-activated receptor gamma coactivator a (PGC-1a) is a transcriptional modulator which regulates the expression of genes contributing to mitochondrial metabolism, biogenesis and oxidative stress [43]. SIRT1 activates PGC-1a, which enhances mitochondrial expression of antioxidant genes including GPx, CAT, and SOD [41, 44]. SIRT1 may reduce the cellular ROS load *via* preventing the expression and production of inducible nitric oxide synthase (iNOS) and nitrous oxide (NO) through the deacetylation of p65, leading to the suppression of the nuclear factor-kappaB (NF-kB) signaling pathway [45].

#### 4.1.2. SIRT2

SIRT2 is located in the cytoplasm. Nie *et al.* [38] reported that oxidative stress augments SIRT2 levels in cells and reduction of SIRT2 results in a lower production of H_2_O_2_-induced ROS. Wang *et al.* [39] reported that oxidative stress upregulates SIRT2 expression in cells. Keskin-Aktan *et al.* [46] found in an animal model study that curcumin treatment significantly reduced MDA and SIRT2 expression in the hippocampus of rats, and that SIRT2 expression was positively associated with MDA. Taken together, the protective effect of curcumin against oxidative stress might be attributed to its capacity to increase levels of SIRT1 and reduce levels of SIRT2 [39].

#### 4.1.3. SIRT3/PGC-1a signaling pathway

SIRT3 is another member of the sirtuins family, which is mainly localized in the mitochondrial matrix and controls mitochondrial fatty-acid oxidation [47]. Overexpression of SIRT3 augments the expression of PGC-1a and lowers the production of ROS (Fig. 10). Zhang *et al.* [48] showed that curcumin reduced oxidative stress by decreasing MDA and increasing SOD, GPx and CAT levels in skeletal muscle mitochondria in a rat model of COPD. Curcumin also upregulated mRNA and protein expression of PGC-1a and SIRT3. The authors concluded that curcumin may possibly attenuate oxidative stress by augmenting the PGC-1a/SIRT3 signaling pathway [48].

#### 4.1.4. Keap1-Nri2-ARE signaling pathway

The Keap1-Nrf2-ARE pathway is known to be the main regulator of oxidative and electrophilic stress responses. In this pathway, the nuclear factor erythroid 2-related factor 2 (Nrf2), a transcription factor, binds to the antioxidant response element (ARE) in the regulatory regions of target genes along with small Maf proteins, allowing Kelch ECH associating protein 1 (Keapl) to bind to Nrf2 and repress it [49]. According to the current evidence, activation of the Keap1-Nrf2- ARE signaling pathway by curcumin may possibly diminish oxidative stress. He *et al.* [50] reported that curcumin treatment in mice fed with a high fat diet diminished the expected increase in muscular MDA and ROS and reversed the reduced level of nuclear factor erythroid-related factor-2 (Nrf2) and hemeoxygenase-1 (HO-1, a stress-response protein). Shi *et al.* [51] reported that activation of the Keap1-Nrf2-ARE pathway decreased oxidative stress. Xie *et al.* [52] showed that curcumin treatment in diabetic rats inhibited oxidative stress through elevated expression of CAT, GSH-Px, HO-1 and norvegicus NAD(P)H quinone dehydrogenase 1, while reducing the expression of SOD1. Moreover, curcumin treatment enhanced expression of the Keap1 protein and increased the nuclear accumulation of Nrf2. The authors concluded that oxidative stress may be diminished by curcumin *via* activating the Keap1-Nrf2-ARE signaling pathway [52]. Wicha *et al.* [53], in an animal model study, demonstrated that treating rats with hexa-hydrocurcumin significantly lowered oxidative stress, MDA and NO levels and also enhanced Nrf2 and HO-1 expression, antioxidative enzyme and SOD activity.

#### 4.1.5. Wnt/p-Catenin Signaling Pathway

The Wnt/p-catenin pathway is activated by connecting a Wnt-protein ligand to a Frizzled family receptor and a lipoprotein receptor related protein 6/5 (LRP6 or LRP5), which leads to the accumulation of P-catenin in the nucleus that forms complexes with DNA-bound T cell [54]. Lima *et al.* [55] have shown the Wnt signaling pathway participates in the reduction of oxidative stress and the enhancement of antioxidant activity. Wang *et al.* [56] demonstrated that curcumin treatment leads to higher mRNA and protein expressions of Wnt3a and P-catenin and mRNA expressions of c-myc and cyclinD1 as well as elevated SOD and GSH-Px levels, while lowered level of MDA in rat models of Parkinson disease. The authors concluded that the protective effect of curcumin against oxidative stress is related to the activation of the Wnt/p-catenin signaling pathway.

This study had some limitations. There was heterogeneity in the studies included with regards to population characteristics; the form, dose and time of the curcumin supplementation; and the measurement method of the variables studied. Most of the studies potentially eligible for this review had to be excluded due to inadequate information.

## 5. Conclusion

The findings indicate that supplementation with curcumin was effective in decreasing MDA and improving levels of antioxidants in diseased individuals under conditions of oxidative stress. The reduction of oxidative stress by curcumin supplementation was dependent on the dose of curcumin and the duration of treatment. The administration of piperine with curcumin may enhance the efficacy of curcumin on antioxidant defense system. The findings suggest the use of curcumin as a cheap and safe adjunct therapy in individuals with oxidative-associated metabolic or neurological diseases.
